# Establishing how social capital is studied in relation to cardiovascular disease and identifying gaps for future research—A scoping review protocol

**DOI:** 10.1371/journal.pone.0249751

**Published:** 2021-04-08

**Authors:** Marie Parker, Xiangming Fang, Shannon Renee Self-Brown, Ali Rahimi

**Affiliations:** 1 School of Public Health, Georgia State University, Atlanta, GA, United States of America; 2 Cardiology, The Southeast Permanente Medical Group, Atlanta, GA, United States of America; Icahn School of Medicine at Mount Sinai, UNITED STATES

## Abstract

**Introduction:**

Though the relationship between social capital and health has been widely studied, the evidence of this relationship in cardiovascular disease is limited, with varied and inconsistent measures. This scoping review seeks to address this gap by answering the following questions: (1) How has social capital been characterized and measured in the literature related to cardiovascular disease? and (2) What gaps exist in the evaluation of the relationship between social capital and cardiovascular disease?

**Materials and methods:**

A scoping review will be used to answer the research questions. The scoping review will apply established methods described by Arksey and O’Malley, Levac and colleagues, and the Joanne Briggs Institute: (1) identifying the research question(s); (2) identifying relevant studies; (3) selecting the studies; (4) charting the data; and (5) collating, summarizing, and reporting the results.

**Results:**

Our findings will be reported in accordance with the guidance provided in the Preferred Reporting Items for Systematic Reviews and Meta-Analyses Protocols (PRISMA-P) statement.

**Discussion:**

The synthesis of this evidence base is intended to provide a framework for how social capital has been defined and measured in the cardiovascular literature, with additional guidance for future research and evaluation. The findings will be disseminated through peer-reviewed publication and presentations at relevant seminars.

## Introduction

Since the Institute of Medicine highlighted the connection between economic and social conditions and the American disparity in morbidity and mortality rates compared to its international counterparts, the role of social determinants of health as drivers of health outcomes in the United States has drawn significant attention [1]. Social determinants of health, as defined by the World Health Organization, encompass the conditions in which individuals work, live, and play and are influenced by money, power, and other resources that include beneficial social connections [2]. The concept of beneficial social connections is most succinctly captured in the construct, social capital, or the benefit one receives from their connections with others. However, there is a lack of consensus on the operational definition of social capital [3].

It is widely accepted that social capital theory was brought into contemporary times by Pierre Bourdieu, James Coleman, and Robert Putnam [4–6]. An exhaustive review of the theories’ nuances is beyond the scope of this review; however, it is important to note that all three authors agree that social capital is comprised of cognitive and structural elements [4–6]. These elements are connected and mutually reinforcing, while each contributes uniquely to social capital, as a whole. Cognitive social capital, referred to as social support henceforth, represents cohesion while structural social capital, hereafter referred to as social network, represents ties.

There is a wealth of literature linking the multidimensional construct of social capital and health, primarily using support and network indicators [3]. Within this body of evidence, the measures of social capital, social support, and social network are varied and inconsistent. As it relates to chronic disease, specifically cardiovascular disease, the evidence is both limited and the measure of social capital is varied [7].

To our knowledge, there is no review that has sought to illustrate the various ways in which social capital, in and of itself, or as a function of support or networks, has been described and measured in the context of health or, more specifically, cardiovascular disease. To that end, a scoping review to understand the relationship of social capital and its concepts and associations with cardiovascular disease will be conducted. The specific purpose of this review is two-fold. First, the review will summarize how social capital and its cognitive and structural concepts, social support and social networks, respectively, are defined, characterized, and measured in the cardiovascular literature. Next, this review will identify themes as well as author, journal, and keyword co-occurrence trends and research gaps about the relationship between social capital and risk factors, incidence, and outcomes of chronic cardiovascular disease to help inform future research opportunities.

## Materials and methods

Scoping reviews are an emerging research methodology, like systematic reviews in their structured approach but different in their purpose and methodologies [8]. Because our study aims are broad, to understand the characterization and methods used to examine the relationship between social capital and cardiovascular disease, reporting and mapping these findings to inform future research in this area, a scoping review was deemed more appropriate than a systematic review [8]. The scoping review will be guided by the methodological framework first proposed by Arksey and O’Malley [9], later refined by Levac et al. [10], and further outlined in the Joanna Briggs Institute Reviewer’s Manual [11]. The stages of the review include (1) identifying the research question; (2) identifying the relevant studies; (3) study selection; (4) charting the data; and (5) collating, summarizing, and reporting the results; and will be described in detail below.

This protocol has been registered within the Open Science Framework (osf.io/s7dpv) and will be reported in accordance with the guidance provided in the Preferred Reporting Items for Systematic Reviews and Meta-Analyses Protocols (PRISMA-P) statement [12] (S1 Checklist).

### Stage 1: Identifying the research question

The PCC (Population–Concept–Context) framework was used to identify the main concepts in the primary review question and inform the search strategy (Table 1) [11]. The primary review question will be *“How has social capital been characterized and measured in the literature related to cardiovascular disease*?*”* to explore how social capital and its components are used in this evaluative body of literature. A sub-question to this will be “*What gaps exist in the evaluation of the relationship between social capital and cardiovascular disease*?*”* to map the range of relevant literature around this relationship and inform the direction of future research.

**Table 1:** PCC framework for identifying the main concepts of the scoping review study.

PCC Element	Definition
**P**opulation	Individuals with cardiovascular disease
**C**oncept	Social Capital, including cognitive and structural components
	Cardiovascular disease, including risk factors, incidence, and outcomes
**C**ontext	Research publications within the last 10 years (2010–2020) and published in English

### Stage 2: Identifying the relevant studies

A thorough search will be conducted in EMBASE, MEDLINE, Web of Science, and Google Scholar [13]. The keyword search strategy will include a combination of subject headings, e.g. Emtree and MEdical Subject Headings (MESH), and Boolean terms, AND/OR, to identify relevant studies. After searching the databases, authors, titles, and abstracts will be imported into a spreadsheet where duplicates will be removed, and screening will be conducted. The reference list of all included studies will also be reviewed for additional relevant studies.

The search will be limited to publications that examine the relationship between social capital or its constructs in humans with cardiovascular disease. Social capital constructs will be identified using social support and related terms to identify cognitive concepts while social network and related terms will be used for structural concepts of social capital. Cardiovascular disease will be limited to those conditions and events commonly associated with chronic disease, e.g. coronary artery disease and myocardial infarction. In addition, studies published within the last 10 years will be included to reflect contemporary definitions of social capital. Lastly, only publications in English will be included as the resources to translate studies in other languages are not available. The full initial search strategy is provided in Table 2.

**Table 2:** Initial search terms and limitations.

Database	Search Terms	Limitations
EMBASE	(’cardiovascular disease’/mj OR ’heart disease’/mj OR ’coronary artery disease’/mj OR ’cardiomyopathy’/mj OR ’heart failure’/mj OR ’heart infarction’/mj)	Human subjects
		English language
		2010–2020 publication years
	AND	
	(’social capital’/mj OR ’social structure’/mj OR ’social network’/mj OR ’social behavior’/mj OR ’social support’/mj OR ’social isolation’/mj)	
MEDLINE / Pubmed	("Cardiovascular Diseases"[Majr] OR "Heart Diseases"[Majr] OR "Coronary Artery Disease"[Majr] OR "Cardiomyopathies"[Majr] OR "Heart Failure"[Majr] OR "Myocardial Infarction"[Majr])	Human subjects
		English language
		2010–2020 publication years
	AND	
	(social environment[Majr] OR social networking[Majr] OR social isolation[Majr] OR social norms[Majr] OR social capital[Majr])	
Web of Science	(TI = ("cardiovascular disease" OR "heart disease" OR "coronary artery disease" OR "cardiomyopathy" OR "heart failure" OR "myocardial infarction"))	English language
		2010–2020 publication years
	AND	
	TI = (("social capital" OR "social network" OR "social support" OR "social integration" OR "social isolation"))	
Google Scholar	allintitle:" social" AND "cardiovascular disease"	English language
		2010–2020 publication years

### Stage 3: Study selection

#### Inclusion criteria

In addition to the parameters of the search strategy “Table 2”, studies will be included if they meet the following criterion:

- Published, peer-reviewed research articles
- The test of association is between social capital or one of its constructs and risk factors, incidence, or outcomes related to cardiovascular disease

#### Exclusion criteria

Studies will be excluded if they meet the following criterion:

- Studies where the outcome of interest is a cardiovascular procedure, e.g. coronary artery bypass graft or percutaneous coronary intervention
- Studies where no abstract is available or where full-text articles cannot be obtained
- Book chapters, commentaries, erratum, meeting abstracts

All identified publications will undergo two stages of screening: title/abstract screening and full text screening. The primary reviewers (MP and XF) will complete the initial screening of all titles and abstracts against the inclusion criteria, indicating whether the publication meets inclusion criteria or, in the event of uncertainty, communicating with the other reviewers (SSB, AR) to clarify inclusion and exclusion criteria. Any titles/abstracts that cannot be definitively included or excluded will be evaluated by another reviewer (AR). The primary reviewers (MP and XF) will then evaluate all full-text publications and a 10% random sample of full-text publications will be evaluated by another reviewer (SSB) to validate the final inclusion decision. Intra- and inter-rater reliability will be assessed using Cohen’s kappa coefficient (x) statistic. To minimize recall bias in the intra-rater reliability assessment, one of the primary reviewers (MP) will conduct the repeated screenings 14–21 days after the first screenings.

### Stage 4: Charting the data

Data from included full-text studies will be entered into a Microsoft Excel form and include the following information:

- Author(s) and year of publication
- Study title and aim(s) or objective(s)
- Study methodology(ies)
- Social capital construct and measure
- Cardiovascular disease and outcome
- Most significant finding(s) and conclusion(s)

Two reviewers (XF, SSB) will review a 10% sample of the final full-text publications to validate the consistency and accuracy of the data charting form, and any discrepancies will be further adjudicated by a third reviewer (AR). Once the final data charting form is agreed upon by all reviewers (MP, XF, SSB, AR), one of the primary reviewers (MP) will complete data extraction for the remaining full-text studies.

## Results

### Stage 5: Collating, summarizing, and reporting the results

A flowchart will be used to detail the study selection process [14]. Three distinct steps will be followed in this stage of the scoping process: analyzing the data, reporting the results, and applying meaning to the results [10]. Data analyses will include a thematic evaluation to highlight social capital and cardiovascular disease themes within the included studies and a bibliometric analysis to report author, journal, and keyword co-occurrence trends. A systematic evaluation of study quality or risk of bias will not be included, as the intent of this scoping review is to provide an overview of the existing literature rather than a critical appraisal of the literature to answer a specific question [8, 9, 14]. The suitability of various formats to present results such as charts, maps, narrative summaries, or tables will be assessed based on the type of data charted from the full-text studies. The meaning of these results will then be considered as they relate to the primary review question, the characterization and measurement of social capital related to cardiovascular disease, and its sub-question regarding existing evidentiary gaps and, subsequently, the implications for future research.

## Discussion

The aim of our work is to identify how social capital and its relationship to cardiovascular disease has been evaluated and highlight knowledge gaps in this area. We will conduct a scoping review that includes a comprehensive literature search across multiple electronic databases, the use of a mixed methods approach to chart and map findings, and a discussion of knowledge gaps and future research opportunities.

There are two primary ways in which the proposed study described within this protocol differs from other methods and protocols. The first is that we use a relatively new literature review methodology, the scoping review. A scoping review is more appropriate for our study than the more common systematic review because our research questions are broad and seek to provide an overview of the literature. The next is that we combine qualitative and quantitative methods to report our results, using thematic themes and bibliometric trends to present our findings in a systematic manner which are intended to provide efficient guidance and meaningful insights into this research area.

The strength of our approach is in the application of new and varied methods to review the literature and report our results. To our knowledge, this will be the first such study that combines these methods in a way to look specifically at social capital and cardiovascular disease. Similarly, the limitations in this study can be attributed to our choice of literature review methodology. Scoping reviews focus on mapping the breadth of studies rather than the depth of information, describe what is known rather than contributing new knowledge, and do not generally include an assessment of study quality or bias.

The review will provide a framework from which future evaluations may draw common social capital definitions and themes and upon which cardiovascular disease risk and outcomes research can build. We expect that our findings will be of interest to researchers and practitioners who are concerned with the intersection of social capital and cardiovascular disease, and plan to disseminate them through peer-reviewed publication and seminar presentations.

## Supporting information

S1 Checklist PRISMA-P (Preferred Reporting Items for Systematic review and Meta-Analysis Protocols) checklist. (DOC)
